# Supplementary material for: Are ELISA and PCR Discrepancies in the Identification of Chlamydia pneumoniae Caused by the Presence of “Chlamydia-Related Bacteria”?
Source: Microorganisms. 2023 Jan 11;11(1):187. doi: 10.3390/microorganisms11010187 (PMC9865915; doi:10.3390/microorganisms11010187)
Supplement: Supplementary file 1 [file microorganisms-11-00187-s001.zip › microorganisms-2145907-supplementary.pdf]

```
                                                                           Panout
                                                                         RYGGRGAAA
Protoch.naegleriophila            AACCTCGGAACAGCATTTGAAACTACATCTCTCGAGGGTAGGCGGAGAAA
Ca.Protoch.amoebophila            AACCTCGGAACAGCATTTGAAACTACATCTCTAGAGGGTAGGCGGAGAAA
Parach.acanthamoebae              AACTCCAGAACAGCATTTGACACTATATTTCTTGAGGGTAGGCGGAGAAA
Ca.Metachlamydia                  AACCCCGGAACAGCATTTGAAACTCCATTTCTAGAGGGTAGGCGGAGAAA
Ca.Mesoch.elodeae                 AACTTCGGAACAGCATTTGAAACTCCCTTTCTAGAGGGTAGGCGGAGAAA
Ca.Rubidus_massiliensis           AACCTCGGAGCTGCATAGGAAACTTCTTGTCTAGAGGGTAGGCGGAGAAA
Neoch.hartmannellae               AACCCCGGAACAGCATTTGAAACTCCCTTTCTTGAGGGTAGACGGAGAAA
Can.Fritschea_bemisiae            AACTTCGAAGCTGCATTTGAAACTGCTTGTCTAGAGGGTAGACGGAGAAA
Ca.Fritschea_eriococci            AACTTCGAAGCTGCATTTGAAACTGCTTGTCTAGAGGATAGACGGAGAAA
Ca.Syngnamydia                    AACTTCGAAGCTGCATTTGAAACTATTTGTCTTGAGGGTAGACGGAGAAA
Ca.Neptunoch.vexilliferae         AACTTCGAAGCTGCATTTGAAACTACTAATCTTGAGGGTAGACGGAGAAA
Ca.Syngnamydia_venezia            AACTTCGAAGCTGCATTTAAAACTACTGATCTTGAGGGTAGACGAAGAAA
Simkania_negevensis               AACTCCGGAGCTGCATTTGAAACTACTTATCTTGAGGGTGGACGGAGAAA
Rhabdoch.crassificans             AACTTCGGAGCCGCATTTGAAACTGCAATTCTTGAGGAATGGCGGAGGAA
Ca.Rhabdoch.porcellionis          AACTTTGGAGCCGCATTTGAAACTGCAATTCTTGAGGGATGGCGGAGGAA
Ca.Renich.lutjani                 AACCCCGGAGCTGCATATTAAACTATATTTATAGAGGGATGGCGGAGAAA
Waddlia_sp.G817                   AACCCCGGAACTGCGTTTGAAACTATATTTCTTGAGGCAAGGCGGAGAAA
Waddlia_chondrophila              AACCCCGGAACTGCGTTTGAAACTATATTTCTAGAGGCAAGGCGGAGAAA
Estrella                          AACCTCAGGGCGGCACCTGAAACTATTTGTCTAGAGGGTAGGCGGAGAAA
Criblamydia_sequanensis           AACCTCGGAACGGCACTCGAAACTGTGTTTCTAGAGGATAGAAGGAGAAA
Ch.sanzinia                       AACCCCAAGTCAGCACTTAATACTGCTTTTCTAGAGGTTAGATGGAGAAA
Ch.ibidis                         AACTCCAAACAGGCATCTAATACTATCTTTCTAGAGGTTAGATGGAGAAA
Ch.pneumoniaeAR39                 AACCCCAAGTCAGCATTTAAAACTATCTTTCTAGAGGATAGATGGGGAAA
Ch.pecorum                        AACCTCAAGTCGGCATCTAATACTATCTTTCTAGAGGTTGGATGGAGAAA
Ch.felis_strain                   AACCCCAAATCGGCATCTAATACTATCTTTCTAGAGGGTAGATGGAGAAA
Ch.caviae                         AACCCCAAGCCAGCATCTAATACTATCTTTCTAGAGGGTAGATGGAGAAA
Ch.psittaci                       AACCCCAAGCCAGCATCTAATACTATCTTTCTGGAGGGTAGATGGAGAAA
Ch.gallinacea_08-1274/3           AACTTCAAGCCGGCATCTAATACTATCTTTCTAGAGGGTAGATGGAGAAA
Ch.avium_strain                   AACCCTAAGTCAGCATCTAATACTATCTTTCTAGAGGGTAGATGGAGAAA
Ch.suis                           AACCCCGAATCGGCATCTGATACTATTTTTCTAGAGGGTAGATGGAGAAA
Ch.muridarum                      AACCCCGAATCGGCATCTAAAACTATTTTTCTAGAGGGTAGATGGAGAAA
Ch.trachomatis                    AACCCCGAGTCGGCATCTAATACTATTTTTCTAGAGGATAGATGGAGAAA
Ca.Amphibiich.                    AACTCTAAGTCAGCATCTAATACTGCCTATCTAGAGGACAGATGGAGAAA
Ca.Amphibiich.ranarum_strain      AACTTCAAGTCAGCATCTAATACTATCTATCTAGAGGACAGACGGAGAAA
Ca.Clavochl.salmonicola           AACCCCGAACAGGCATCTAAAACTGTATTTCTAGAGGTCGGGAGGAGAAA
Ca.Similich.latridicola           ATCTCCGGA-CTGCATACAAAACTGCTCAGCTAGAGTATAAGAGGGGAAA
Ch.bacterium_clone                ATCTTCGGA-CTGCATACAAAACTGCTTGACTAGAGTGTAAGAGGGGAAA
Ca.Similich.labri                 ATCTCCGGA-CTGCATACAAAACTGTTCAGCTAGAGCATAAGAGGGGAAA
Ch.bacterium_CF280911-8           ATCTCCGGA-TCGCATACAAAACTGCTTGACTAGAGTGTAATAGGGGAAA
Ca.Piscichl._salmonis             ACCCCCAGG-CTGCTTCCAAAACTGCTAGACTAGAGTTCAAGAGGGGGAA
                                  * *         **     * ***       * ***       *  * **
                                                                         RYGGRGAAA

                                    Panout
                                  RNGGAATTCCA
Protoch.naegleriophila            ACGGAATTCCACAAGTAGC-GGTGAAA-TGCGTAGATATGTGGAAGAACA
Ca.Protoch.amoebophila            ACGGAATTCCACAAGTAGC-GGTGAAA-TGCGTAGATATGTGGAAGAACA
Parach.acanthamoebae              ACGGAATTCCACATGTAGC-GGTGAAA-TGCGTAGATATGTGGAAGAACA
Ca.Metachlamydia                  ACGGAATTCCACAAGTAGC-GGTGAAA-TGCGTAGATATGTGGAAGAACA
Ca.Mesoch.elodeae                 ACGGAATTCCACAAGTAGC-GGTGAAA-TGCGTAGATATGTGGAAGAACA
Ca.Rubidus_massiliensis           ATGGAATTCCACAAGTAGC-GGTGAAA-TGCGTAGATATGTGGAAGAACA
Neoch.hartmannellae               ATGGAATTCCACAAGTAGC-GGTGAAA-TGCGTAGATATGTGGAAGAACA
Can.Fritschea_bemisiae            ACGGAATTCCAAGTGTAGC-GGTGAAA-TGCGTAGATATTTGGAAGAACA
Ca.Fritschea_eriococci            ACGGAATTCCAAGTGTAGC-GGTGAAA-TGCGTAGATATTTGGAAGAACA
Ca.Syngnamydia                    ACGGAATTCCAAGTGTAGC-GGTGAAA-TGCGTAGATATTTGGAAGAACA
Ca.Neptunoch.vexilliferae         ACGGAATTCCAAGTGTAGC-GGTGGAA-TGCGTAGATATTTGGAAGAACA
Ca.Syngnamydia_venezia            ATGGAATTCCAAGTGTAGC-GGTGGAA-TGCGTAGATATTTGGAAGAACA
Simkania_negevensis               ACGGAATTCCACGTGTAGC-GGTGAAA-TGCGTAGATATGTAGAAGAACA
Rhabdoch.crassificans             ACGGAATTCCACGTGTAGC-GGTGAAA-TGCTTAGATATGTGGAAGAACA
Ca.Rhabdoch.porcellionis          ACGGAATTCCACGTGTAGC-GGTGAAA-TGCGTAGATATGTGGAAGAACA
Ca.Renich.lutjani                 ATGGAATTCCACGTGTAGC-GGTGAAA-TGCGTAGAAATGTGGAAGAACA
Waddlia_sp.G817                   ACGGAATTCCACATGTAGC-GGTGAAA-TGCGTAGATATGTGGAAGAACA
Waddlia_chondrophila              ACGGAATTCCCCATGTTGCCGGTGAAAATGCGTAGATATTTGGAAAAACA
Estrella                          ACGGAATTCCACAAGTAGC-GGTGAAA-TGCGTAGATATGTGGAAGAACA
Criblamydia_sequanensis           ATGGAATTCCACAAGTAGC-GGTGAAA-TGCGTAGATATGTGGAAGAACA
Ch.sanzinia                       AGGGAATTCCACATGTAGC-GGTGAAA-TGCGTAGATATGTGGAAGAACA
Ch.ibidis                         AGGGAATTCCACGTGTAGC-GGTGAAA-TGCGTAGATATGTGGAAGAACA
Ch.pneumoniaeAR39                 AGGGAATTCCACGTGTAGC-GGTGAAA-TGCGTAGATATGTGGAAGAACA
Ch.pecorum                        AGGGAATTCCACGTGTAGC-GGTGAAA-TGCGTAGATATGTGGAAGAACA
Ch.felis_strain                   AGGGAATTCCACGTGTAGC-GGTGAAA-TGCGTAGATATGTGGAAGAACA
Ch.caviae                         AGGGAATTCCACGTGTAGC-GGTGAAA-TGCGTAGATATGTGGAAGAACA
Ch.psittaci                       AGGGAATTCCACGTGTAGC-GGTGAAA-TGCGTAGATATGTGGAAGAACA
Ch.gallinacea_08-1274/3           AGGGAATTCCACGTGTAGC-GGTGAAA-TGCGTAGATATGTGGAAGAACA
Ch.avium_strain                   AGGGAATTCCACGTGTAGC-GGTGAAA-TGCGTAGATATGTGGAAGAACA
Ch.suis                           AGGGAATTTCACGTGTAGC-GGTGAAA-TGCGTAGATATGTGGAAGAACA
```

```
Ch.muridarum                      AGGGAATTTCACGTGTAGC-GGTGAAA-TGCGTAGATATGTGGAAGAACA
Ch.trachomatis                    AGGGAATTTCACGTGTAGC-GGTGAAA-TGCGTAGATATGTGGAAGAACA
Ca.Amphibiich.                    AGGGAATTCCACGTGTAGC-GGTGAAA-TGCGTAGATATGTGGAAGAACA
Ca.Amphibiich.ranarum_strain      AGGGAATTCCACATGTAGC-GGTGAAA-TGCGTAGATATGTGGAAGAACA
Ca.Clavochl.salmonicola           AGGGAATTCCACGTGTAGC-GGTGAAA-TGCGTAGATATGTGGAAGAACA
Ca.Similich.latridicola           GCGGAATTCCACAAGTAGC-GGTGAAA-TGCGTAGATATGTGGAGGAACA
Ch.bacterium_clone                GCGGAATTCCACAAGTAGC-GGTGAAA-TGCGTAGATATGTGGAGGAACA
Ca.Similich.labri                 GCGGAATTCCACAAGTAGC-GGTGAAA-TGCGTAGATATGTGGAGGAACA
Ch.bacterium_CF280911-8           GCGGAATTCCACAAGTAGC-GGTGAAA-TGCGTAGATATGTGGAGGAACA
Ca.Piscichl._salmonis             ATGGAATTCCACAAGTAGC-GGTGAAA-TGCGTAGATATGTGGAGGAACA
                                    ****** *     ** ** **** ** *** **** ** * **  ****
                                  RNGGAATTCCA

                                           Panin
                                     GTGGCGAAGGCGCTTTC
Protoch.naegleriophila            CCGGTGGCGAAGGCGGTTTTCTAGCTTATTCCTGACGCTAAAGCGCGAAA
Ca.Protoch.amoebophila            CCGGTGGCGAAGGCGGTTTTCTAGCTTATACCTGACGCTAAAGCGCGAAA
Parach.acanthamoebae              TCGGTGGCGAAGCGGGTTTTCTAGCTTATTCCTGACGCTGAGGCGCGAAA
Ca.Metachlamydia                  CCGGTGGCGAAGGCGGTTTTCTAGCTTATTCCTGACGCTGAGGCGCGAAA
Ca.Mesoch.elodeae                 CCGGTGGCGAAGGCGGTTTTCTAGCTTATACCTGACGCTGAGGCGCGAAA
Ca.Rubidus_massiliensis           CCAGTGGCGAAGGCGGTTTTCTAGCTTATACCTGACGCTGAGGCGCGAAA
Neoch.hartmannellae               CCAGTGGCGAAGGCGATTTTCTAGTTTACACCTGACGCTGAGGCGCGAAA
Can.Fritschea_bemisiae            CCGGTGGCGAAAGCGGTTTTCTAGTTTATTCCTGACGCTGAGATGCGAAA
Ca.Fritschea_eriococci            CCGGTGGCGAAAGCGGTTTTCTAGTTTATTCCTGACGCTGAGATGCGAAA
Ca.Syngnamydia                    CCGGTGGCGAAAGCGGTTTTCTAGTTTATTCCTGACGCTGAGATGCGAGA
Ca.Neptunoch.vexilliferae         CCGGTGGCGAAAGCGGTTTTCTAGTTTATTCCTGACGCTGAGACGCGAGA
Ca.Syngnamydia_venezia            CCGGTGGCGAAAGCGGTTTTCTAGTTTATTCCTGACGCTGAGATGCGAGA
Simkania_negevensis               CCGGTGGCGAAGGCGGTTTTCTAGTTCTTTCCTGACGCTGAGGCGCGAGA
Rhabdoch.crassificans             CCTGTGGCGAAAGCGGTTTTCTAGCTTTTACCTGACGCTGAGGCGCGAGA
Ca.Rhabdoch.porcellionis          CCTGTGGCGAAAGCGGTTTTCTAGCTTTTACCTGACGCTGAGGCGCGAGA
Ca.Renich.lutjani                 CCAGTGGCGAAAGCGGTTTTCTTGTTATTTCCTGACGCTGAGGGGGGAGA
Waddlia_sp.G817                   CCAGTGGCGAAAGCGGTTTTCTAGCTTTGACCTGACGCTGATGCCCGAAA
Waddlia_chondrophila              CCAGTGGCGAAGGCGGTTTTCTAGCTTTGACCTGACGCTGATGCCCGAAA
Estrella                          CCGGTGGCGAAGGCGGTTTTCTAGCTTACTCCTGACGCTGAGGCGCGAAA
Criblamydia_sequanensis           CCGGTGGCGAAGGCGGTTTTCTATTTTATTCCTGACGCTCATGCACGAAA
Ch.sanzinia                       CCAGTGGCGAAGGCGCTTTTCTAATTTATTCCTGACGCTAAGGCGCGAAA
Ch.ibidis                         CCAGTGGCGAAGGCGCTTTTCTAATTTATTCCTGACGCTGAGGCGCGAAA
Ch.pneumoniaeAR39                 CCAGTGGCGAAGGCGCTTTTCTAATTTATACCTGACGCTAAGGCGCGAAA
Ch.pecorum                        CCAGTGGCGAAGGCGCTTTTCTAATTTATTCCTGACGCTAAGGCGCGAAA
Ch.felis_strain                   CCAGTGGCGAAGGCGCTTTTCTAATTTACACCTGACGCTAAGGCGCGAAA
Ch.caviae                         CCAGTGGCGAAGGCGCTTTTCTAATTTACACCTGACGCTAAGGCGCGAAA
Ch.psittaci                       CCAGTGGCGAAGGCGCTTTTCTAATTTACACCTGACGCTAAGGCGCGAAA
Ch.gallinacea_08-1274/3           CTAGTGGCGAAGGCGCTTTTCTAATTTACACCTGACGCTGAGGCGCGAAA
Ch.avium_strain                   CTAGTGGCGAAGGCGCTTTTCTAATTTACACCTGACGCTAAGGCGCGAAA
Ch.suis                           CCAGTGGCGAAGGCGCTTTTCTAATTTATACCTGACGCTAAGGCGCGAAA
Ch.muridarum                      CCAGTGGCGAAGGCGCTTTTCTAATTTATACCTGACGCTAAGGCGCGAAA
Ch.trachomatis                    CCAGTGGCGAAGGCGCTTTTCTAATTTATACCTGACGCTAAGGCGCGAAA
Ca.Amphibiich.                    CCAGTGGCGAAGGCGCTTTTCTAATTTGGACCTGACGCTGAGGCGCGAAA
Ca.Amphibiich.ranarum_strain      TCAGTGGCGAAGGCGCTTTTCTAGTTTGATCCTGACGCTGAGGCGCGAAA
Ca.Clavochl.salmonicola           CCGGTGGCGAAGGCGCTTTTCTATCTTGATCCTGACGCTGAGGCGCGAAA
Ca.Similich.latridicola           CCGGTGGCGAAGGCGGCTTTCTGGATTATTACTGACGCTGAGGCACGAAA
Ch.bacterium_clone                CCGGTGGCGAAGGCGGCTTTCTGGATTATTACTGACGCTGAGGCACGAAA
Ca.Similich.labri                 CCGGTGGCGAAGGCGGCTTTCTGGATTATTGCTGACGCTGAGGCACGAAA
Ch.bacterium_CF280911-8           CCAGTGGCGAAGGCGGCTTTCTGGGTTACTACTGACGCTGAGGCACGAAA
Ca.Piscichl._salmonis             CCGGTGGCGAAGGCGGTTTCCTAGATTGATACTGACGCTGAGGCACGAAA
                                     ********    *  ** **    *      ******** *      ** *
                                     GTGGCGAAGGCGCTTTTC

Protoch.naegleriophila            GCAAGGGG-ATCAAACAGGATTAGATACCCTGGTAGTCCTTGCCGTAAAC
Ca.Protoch.amoebophila            GCAAGGGG-ATCAAACAGGATTAGATACCCTGGTAGTCCTTGCCGTAAAC
Parach.acanthamoebae              GCAGGGGGAGCAAACAGGATTAGATACCCTGGTAGTCCTTGCCGTAAAC
Ca.Metachlamydia                  GCAAGGGG-ATCAAACAGGATTAGATACCCTGGTAGTCCTTGCCGTAAAC
Ca.Mesoch.elodeae                 GCAAGGGG-AGCAAACAGGATTAGATACCCTGGTAGTCCTTGCCGTAAAC
Ca.Rubidus_massiliensis           GCAAGGGG-AGCAAACAGGATTAGATACCCTGGTAGTCCTTGCCGTAAAC
Neoch.hartmannellae               GCAAGGGG-ATCAAACAGGATTAGATACCCTGGTAGTCCTTGCCCTAAAC
Can.Fritschea_bemisiae            GCAAGGGG-AGCAAACAGGATTAGATACCCTGGTAGTCCTTGCTGTAAAC
Ca.Fritschea_eriococci            GCAAGGGG-AGCAAACAGGATTAGATACCCTGGTAGTCCTTGCTGTAAAC
Ca.Syngnamydia                    GCAAGGGT-AGCAAACAGGATTAGATACCCTGGTAGTCCTTGCCGTAAAC
Ca.Neptunoch.vexilliferae         GCAAGGGG-AGCAAACAGGATTAGATACCCTGGTAGTCCTTGCCGTAAAC
Ca.Syngnamydia_venezia            GCAAGGGG-AGCAAACAGGATTAGATACCCTGGTAGTCCTTGCCGTAAAC
Simkania_negevensis               GCAAGGGG-AGCAAACAGGGTTAGATACCCTGGTAGTCCTTGCTGTAAAC
Rhabdoch.crassificans             GCAAGGGG-AGCAAACAGGATTAGATACCCTGGTAGTCCTTGCCGTAAAC
Ca.Rhabdoch.porcellionis          GCAAGGGG-AGCAAACAGGATTAGATACCCTGGTAGTCCTTGCCGTAAAC
Ca.Renich.lutjani                 GCAAGGGG-GGCAAACAGGATTAGATACCCTCGTAGTCCCGGCAATAAAC
Waddlia_sp.G817                   GCTAGGGG-AGCAAACAGGATTAGATACCCTGGTAGTCCTAGCCGTAAAC
Waddlia_chondrophila              GCTAGGGG-AGCGAACAGGATTAGATACCCTGGTAGTCCTAGCCGTAAAC
Estrella                          GTTAGGGG-ATCAAACAGGATTAGATACCCTGGTAGTCCTAACCGTAAAC
```

```
Criblamydia_sequanensis          GTTAGGGG-ATCAAACAGGATTAGATACCCTGGTAGTCCTAACTGTAAAC
Ch.sanzinia                      GC-AAGGGGAGCAAACAGGATTAGATACCCTGGTAGTCCTTGCCGTAAAC
Ch.ibidis                        GC-AAGGGGAGCAAACAGGATTAGATACCCTGGTAGTCCTTGCCGTAAAC
Ch.pneumoniaeAR39                GC-AAGGGGAGCAAACAGGATTAGATACCCTGGTAGTCCTTGCCGTAAAC
Ch.pecorum                       GC-AAGGGGAGCAAACAGGATTAGATACCCTGGTAGTCCTTGCTGTAAAC
Ch.felis_strain                  GC-AAGGGGAGCAAACAGGATTAGATACCCTGGTAGTCCTTGCCGTAAAC
Ch.caviae                        GC-AAGGGGAGCAAACAGGATTAGATACCCTGGTAGTCCTTGCCGTAAAC
Ch.psittaci                      GC-AAGGGGAGCAAACAGGATTAGATACCCTGGTAGTCCTTGCCGTAAAC
Ch.gallinacea_08-1274/3          GC-AAGGGGAGCAAACAGGATTAGATACCCTGGTAGTCCTTGCTGTAAAC
Ch.avium_strain                  GC-AAGGGGAGCAAACAGGATTAGATACCCTGGTAGTCCTTGCCGTAAAC
Ch.suis                          GC-AAGGGGAGCAAACAGGATTAGATACCCTGGTAGTCCTTGCCGTAAAC
Ch.muridarum                     GC-AAGGGGAGCAAACAGGATTAGATACCCTGGTAGTCCTTGCCGTAAAC
Ch.trachomatis                   GC-AAGGGGAGCAAACAGGATTAGATACCCTGGTAGTCCTTGCCGTAAAC
Ca.Amphibiich.                   GC-AAGGGGAGCAAACAGGATTAGATACCCTGGTAGTCCTTGCTGTAAAC
Ca.Amphibiich.ranarum_strain     GCCAAGGGGAGCAAACAGGATTAGATACCCTGGTAGTCCTTGCTGTAAAC
Ca.Clavochl.salmonicola          GC-AAGGGGAGCAAACAGGATTAGATGCCCTGGTAGTCCTTGCCGTAAAC
Ca.Similich.latridicola          GCATGGGG-AGCAAACAGGATTAGATACCCTGGTAGTCCATGCCGTAAAC
Ch.bacterium_clone               GCATGGGG-AGCAAACAGGATTAGATACCCTGGTAGTCCATGCCGTAAAC
Ca.Similich.labri                GCATGGGG-AGCAAACAGGATTAGATACCCTGGTAGTCCATGCCGTAAAC
Ch.bacterium_CF280911-8          GCATGGGG-AGCAAACAGGATTAGATACCCTGGTAGTCCATGCCGTAAAC
Ca.Piscichl._salmonis            GCAAGGGG-AGCAAACAGGATTAGATACCCTGGTAGTCCTTGCTGTAAAC
                                 *    **    * ****** ****** **** *******   *  *****

Protoch.naegleriophila           TATGTATACTTGGTGTAACTGGACTCAACCCTAGTTGTGCCGTAGCTAAC
Ca.Protoch.amoebophila           TATGTATACTTGGTGTAACTGGACTCAACCCTAGTTGTGCCGTAGCTAAC
Parach.acanthamoebae             GATGTATACTTGGTGTAATTGGACTCAACCCCAATTGTGCCGGACGTAAC
Ca.Metach.                       GATGTATACTTGATGTAATTGGACTCAACCCCGATTGTGTCGTAGCTAAC
Ca.Mesoch.elodeae                GATGTATACTTGGTGTAGTTGGAATCAACCCCGACTGTGCCGTAGCTAAC
Ca.Rubidus_massiliensis          GATGTATACTTGGTGTAGTTGGACTCAACCCCGACTGTGCCGTAGCTAAC
Neoch.hartmannellae              TACGTATACTTGGTGTAACTGGAATCAACCCTAGTTGTGCCGAACGTAAC
Can.Fritschea_bemisiae           GATGCATACTTGGTGTAGTTGGACTCAACCCCGACTGTGCCGTAGCTAAC
Ca.Fritschea_eriococci           GATGCATACTTGGTGTAGTTGGACTCAACCCCGACTGTGCCGTAGCTAAC
Ca.Syngnamydia                   GATGCATACTTGGTGTAGTTGGACTCAACCCCGACTGTGCCGTAGCTAAC
Ca.Neptunoch.vexilliferae        GATGCATACTTGGTGTAGTTGGACTCAACCCCGACTGTGCCGTAGCTAAC
Ca.Syngnamydia_venezia           AATGCATACTTGGTGTAGTTGGGCTCAACCCCGACTGTGCCGTAGCTAAC
Simkania_negevensis              ACTGCATACTTGGTGTAGTCGGTCTCAACCCCGACTGTGCCGTACGTAAC
Rhabdoch.crassificans            GATGTATACTTGATGTAGTTAGGCTCAACCCTGGCTGTGTCGTAGCTAAC
Ca.Rhabdoch.porcellionis         GATGTATACTTGATGTAGTTAGGCTCAACCCTGGCTGTGTCGTAGCTAAC
Ca.Renich.lutjani                GATGTCTACTTGATGTAGCGGGGCTCAACCCTATCTGTGTCGAAGCCAAC
Waddlia_sp.G817                  GATGCATACTTGGTGTAGCCGGACTCAACCCCGGCTGTGCCGTAGCTAAC
Waddlia_chondrophila             GATGCATACTTGGTGTAGCCGGACTCAACCCTGGCTGTGCCGTAGCTAAC
Estrella                         TATGTATACTTGGTGTAGCTGGTCTCAACCCCAGCTGTGCCGTAGCTAAC
Criblamydia_sequanensis          GATGTGTACTTGGTGTAGCTGGTCTCAACCCCAGCTGTGCCGTAGCCAAC
Ch.sanzinia                      GATGCATACTTGATGTGGATGGTCTCAACCCCATCCGTGTCGGAGCTAAC
Ch.ibidis                        GATGCATACTTGATGTGGATGGTCTCAACCCCATCCGTGTCGGAGCTAAC
Ch.pneumoniaeAR39                GATGCATACTTGATGTGGATGGTCTCAACCCCATCCGTGTCGGAGCTAAC
Ch.pecorum                       GATGCATACTTGATGTGGATGGTCTCAACCCCATCCGTGTCGGAGCTAAC
Ch.felis_strain                  GATGCATACTTGATGTGGATAGTCTCAACCCTATCCGTGTCGTAGCTAAC
Ch.caviae                        GATGCATACTTGATGTGGATAGTCTCAACCCTATCCGTGTCGTAGCTAAC
Ch.psittaci                      GATGCATACTTGATGTGGATAGTCTCAACCCTATCCGTGTCGTAGCTAAC
Ch.gallinacea_08-1274/3          GATGCATACTTGATGTGGATAGTCTCAACCCTATCCGTGTCGTAGCTAAC
Ch.avium_strain                  GATGCATACTTGATGTGGATAGTCTCAACCCTATCCGTGTCGTAGCTAAC
Ch.suis                          GATGCATACTTGATGTGGATGGTCTCAACCCCATCCGTGTCGGAGCTAAC
Ch.muridarum                     GATGCATACTTGATGTGGATGGTCTCAACCCCATCCGTGTCGGAGCTAAC
Ch.trachomatis                   GATGCATACTTGATGTGGATGGTCTCAACCCCATCCGTGTCGGAGCTAAC
Ca.Amphibiich.                   GATGCACACTTGATGTGAGTGGAATCAACCCCGCTCGTGTCGGAGCTAAC
Ca.Amphibiich.ranarum_strain     GATGTACACTTGATGTGAGTGGATTCAACCCCGCTCGTGTCGTAGCTAAC
Ca.Clavochl.salmonicola          GATGCATACTTGGTGTGAGTGGACTCAACCCCACTCGTGCCGGAGCTAAC
Ca.Similich.latridicola          GATGCACACTTGATGTAGTCGGTCTCAACCCCGGCTGTGTCGTAGCTAAC
Ch.bacterium_clone               GATGCACACTTGATGTAGTCGGTCTCAACCCCGGCTGTGTCGTAGCTAAC
Ca.Similich.labri                GATGCACACTTGATGTAGTCGGTCTCAACCCCGGCTGTGTCGTAGCTAAC
Ch.bacterium_CF280911-8          GATGCATACTTGATGTGGACGGTCTCAACCCCGGCCGTGTCGTAGCTAAC
Ca.Piscichl._salmonis            GATGCACACTTGATGTAGCTAGTCTCAACCCTAGCTGTGTCGAAGCTAAC
                                    *   ***** ***     *  *******     *** ** *   ***
                                        ACTTGRTGTRRNYRGWCTCAACC      GTGTYGKAGCTAAC
                                        TGAACYACAYYNRYCWGAGTTGG      CACARCMTCGATTG
                                               PChtin                  Pshort down

Protoch.naegleriophila           GCGTTAAGTATACCGCCTGGGGAGTACAGTCGCAAGGCTGAAACTCAAAA
Ca.Protoch.amoebophila           GCGTTAAGTATACCGCCTGGGGAGTACAGTCGCAAGGCTGAAACTCAAAA
Parach.acanthamoebae             GCGTTAAGTATACCGCCTGGGGAGTACGCTCGCAAGGGTGAAACTCAAAA
Ca.Metachlamydia                 GCGATAAGTATACCGCCTGGGGAGTACGCTCGCAAGGGTGAAACTCAAAA
Ca.Mesoch.elodeae                GCGATAAGTATACCGCCTGGGGAGTACAGTCGCAAGGCTGAAACTCAAAA
Ca.Rubidus_massiliensis          GCGATAAGTATACCGCCTGGGGAGTACGCTCGCAAGGGTGAAACTCAAAA
Neoch.hartmannellae              GCGATAAGTATACCGCCTGAGGAGTACGCTCGCAAGGGTGAAACTCAAAA
Can.Fritschea_bemisiae           GCGTTAAGTATGCCACCTGAGGAGTACGCCCGCAAGGGTGAAACTCAAAA
Ca.Fritschea_eriococci           GCGTTAAGTATGCCACCTGAGGAGTACGCCCGCAAGGGTGAAACTCAAAA
```

```
Ca.Syngnamydia                    GCGTTAAGTATGCCACCTGGGGAGTACGCCCGCAAGGGTGAAACTCAAAA
Ca.Neptunoch.vexilliferae         GCGTTAAGTATGCCACCTGGGGAGTACGCCCGCAAGGGTGAAACTCAAAA
Ca.Syngnamydia_venezia            GTGTTAAGTATGCCACCTGAGGAGTACGCTCGCAAGGGTGAAACTCAAAA
Simkania_negevensis               GCGTTAAGTATGCCACCTGGGGAGTACGCTCGCAAGGGTGAAACTCAAAA
Rhabdoch.crassificans             GCGATAAGTATACCGCCTGAGGAGTACGCTCGCAAGGGTGAAACTCAAAA
Ca.Rhabdoch.porcellionis          GCGATAAGTATACCGCCTGAGGAGTACGCTCGCAAGGGTGAAACTCAAAA
Ca.Renich.lutjani                 GCGATAAGTATACCGCCTGGGGAGTACGCTCCCAGGGGTAAAACTCAAAA
Waddlia_sp.G817                   GCGTTAAGTATGCCGCCTGGGGAGTACGCCCGCAAGGGTAAAACTCAAAA
Waddlia_chondrophila              GCGTTAAGTATGCCGCCTGGGGAGTACGCCCGCAAGGGTAAAACTCAAAA
Estrella                          GCGATAAGTATACCGCCTGGGGAGTACACTCGCAAGGGTGAAACTCAAAA
Criblamydia_sequanensis           GCGTTAAGTACACCGCCTGGGGAGTACAGCCGCAAGGCTGAAACTCAAAA
Ch.sanzinia                       GTGTTAAGTATGCCGCCTGAGGAGTACACTCGCAAGGGTGAAACTCAAAA
Ch.ibidis                         GCGTTAAGTATGCCGCCTGAGGAGTACACTCGCAAGGGTGAAACTCAAAA
Ch.pneumoniaeAR39                 GCGTTAAGTATGCCGCCTGAGGAGTACACTCGCAAGGGTGAAACTCAAAA
Ch.pecorum                        GCGTTAAGTATGCCGCCTGAGGAGTACACTCGCAAGGGTGAAACTCAAAA
Ch.felis_strain                   GCGTTAAGTATGCCGCCTGAGGAGTACACTCGCAAGGGTGAAACTCAAAA
Ch.caviae                         GCGTTAAGTATGCCGCCTGAGGAGTACACTCGCAAGGGTGAAACTCAAAA
Ch.psittaci                       GCGTTAAGTATGCCGCCTGAGGAGTACACTCGCAAGGGTGAAACTCAAAA
Ch.gallinacea_08-1274/3           GCGTTAAGTATGCCGCCTGAGGAGTACACTCGCAAGGGTGAAACTCAAAA
Ch.avium_strain                   GCGTTAAGTATGCCGCCTGAGGAGTACACTCGCAAGGGTGAAACTCAAAA
Ch.suis                           GCGTTAAGTATGCCGCCTGAGGAGTACACTCGCAAGGGTGAAACTCAAAA
Ch.muridarum                      GCGTTAAGTATGCCGCCTGAGGAGTACACTCGCAAGGGTGAAACTCAAAA
Ch.trachomatis                    GCGTTAAGTATGCCGCCTGAGGAGTACACTCGCAAGGGTGAAACTCAAAA
Ca.Amphibiich.                    GCGTTAAGTGTGCCGCCTGAGGAGTACACTCGCAAGGGTGAAACTCAAAA
Ca.Amphibiich.ranarum_strain      GCGTTAAGTGTACCGCCTGAGGAGTACACTCGCAAGGGTGAAACTCAAAA
Ca.Clavochl.salmonicola           GCGTTAAGTATGCCGCCTGAGGAGTACACTCGCAAGGGTGAAACTCAAAA
Ca.Similich.latridicola           GCGATAAGTGTGCCACCTGGGGAGTACGCTCGCAAGGGTGAAACTCAAAA
Ch.bacterium_clone                GCGATAAGTGTGCCACCTGGGGAGTACGCTCGCAAGGGTGAAACTCAAAA
Ca.Similich.labri                 GCGATAAGTGTGCCACCTGGGGAGTACGCTCGCAAGGGTGAAACTCAAAA
Ch.bacterium_CF280911-8           GCGATAAGTATGCCACCTGGGGAGTACGCTCGCAAGGGTGAAACTCAAAA
Ca.Piscichl._salmonis             GCGATAAGTGTGCCACCTGGGGAGTACGCCTGCAAGGGTGAAACTCAAAA
                                  * * *****    ** **** *******      ** ** * **********
                                 GCGTTAAGTATR
                                 CGCAATTCATAY
                                  Pshort down
```

Figure S1. Design of panprimers. Outer primers Panout and Pshort are marked in grey. Internal primers Panin a Pchtin are marked in purple. Degenerated nucleotides are highlighted in red.

```
52CHP                 AGCAAGGGGAGCAAACAGGATTAGATACCCTGGTAGTCCTGGCCGTAAACGATGTATA
3021                  AGCAAGGGGAGCGAACAGGATTAGATACCCTGGTAGTCCTGGCCGTAAACGATGTATA
66SP                  AGCAAGGGGATCAAACAGGATTAGATACCCTGGTAGTCCTTGCCGTAAACTATGTATA
3026                  AGCAAGGGGATCAAACAGGATTAGATACCCTGGTAGTCCTTGCCGTAAACCATGTATA
3033                  AGCAAGGGGATCAAACAGGATTAGATACCCTGGTAGTCCTTGCTGTAAACGATGTATA
27SP                  AGCAAGGGGATCAAACAGGATTAGATACCCTGGTAGTCCTTGCCGTAAACGATGTATA
74CHP                 AGCAAGGGGAGCAAACAGGATTAGATACCCTGGTAGTCCTTGCCGTAAACGATGTATA
109CHP                AGCAAGGGGAGCAAACAGGATTAGATACCCTGGTAGTCCTTGCCGTAAACGATGTATA
2CHP                  AGCAAGGGGAGCAAACAGGATTAGATACCCTGGTAGTCCTTGCCGGAAACGATGCATA
21CHP                 AGCAAGGGGAGCAAACAGGATTAGATACCCTGGYAGTCCTTGCCGTAAACGATGCATA
73CHP                 AGCAAGGGGAGCAAACAGGATTAGATACCCTGGTAGTCCTTGCCGTAAACGATGCATA
110CHP                AGCAAGGGGAGCAAACAGGATTAGATACCCTGGTAGTCCTTGCCGTAAACGATGCATA
11CHP                 AGCAAGGGGAGCAAACAGGATTAGATACCCTGGTAGTCCTTGCCGTAAACGATGCATA
3001                  AGCAAGGGGAGCAAACAGGATTAGATACCCTGGTAGTCCTTGCCGTAAACGATGCATA
19CHP                 AGCAAGGGGAGCAAACAGGATTAGATACCCTGGTAGTCCTTGCCGTAAACGATGCATA
23CHP                 AGCAAGGGGAGCAAACAGGATTAGATACCCTGGTAGTCCTTGCCGTAAACGATGCATA
24CHP                 AGCAAGGGGAGCAAACAGGATTAGATACCCTGGTAGTCCTTGCCGTAAACGATGCATA
31CHP                 AGCAAGGGGAGCAAACAGGATTAGATACCCTGGTAGTCCTTGCCGTAAACGATGCATA
64CHP                 AGCAAGGGGAGCAAACAGGATTAGATACCCTGGTAGTCCTTGCCGTAAACGATGCATA
101CHP                AGCAAGGGGAGCAAACAGGATTAGATACCCTGGTAGTCCTTGCCGTAAACGATGCATA
98CHP                 AGCAAGGGGAGCAAACAGGATTAGATACCCTGGTAGTCCTTGCCGTAAACGATGCATA
36CHP                 AGCAAGGGGAGCAAACAGGATTAGATACCCTGGTAGTCCTTGCCGTAAACGATGCATA
6SP                   AGCAAGGGGAGCAAACAGGATTAGATACCCTGGTAGTCCTTGCCGTAAACGGTGTATA
58SP                  AGCATGGGGAGCAAACAGGATTAGATACCCTGGTAGTCCATGCCGTAAACGATGTATA
81SP                  AGCATGGGGAGCAAACAGGATTAGATACCCTGGTAGTCCATGCCGTAAACGTTGTATA
C. pneumoniae  AR39   AGCAAGGGGAGCAAACAGGATTAGATACCCTGGTAGTCCTTGCCGTAAACGATGCATA
                      **** ***** * ******************** *****  ** * ****  ** ***
```

Figure S2. Alignment of panNPCR 58 nt sequences from sputa. Polymorphic sites are red shadowed. "Chlamydia-related bacteria" are grey shadowed

```
122E               AGCAAGGGGATCAAACAGGATTAGATACCCTGGTAGTCCTTGCCCTAAACTATGTATA
85E                AGCAAGGGGAACAAACAGGATTAGATACCCTGGTAGTCCTTGCCCTAAACTATGTATA
20E                AGCAAGGGGAGCAAACAGGATTAGATACCCTGGTAGTCCTTGCCTTAAACGATGTATA
1E                 AGCAAGGGGAGCAAACAGGATTAGATACCCTGGTAGTCCTTGCCGTAAACGATGCATA
52E                AGCAAGGGGAGCAAACAGGATCAGATACCCTGGTAGTCCTTGCCGTAAACGATGCATA
10E                AGCAAGGGGAGCAAACAGGATTAGATACCCTGGTAGTCCTTGCCGTAAACGATGCATA
38E                AGCAAGGGGAGCAAACAGGATTAGATACCCTGGTAGTCCTTGCCGTAAACGATGCATA
42E                AGCAAGGGGAGCAAACAGGATTAGATACCCTGGTAGTCCTTGCCGTAAACGATGCATA
48E                AGCAAGGGGAGCAAACAGGATTAGATACCCTGGTAGTCCTTGCCGTAAACGATGCATA
6E                 AGCAAGGGGAGCAAACAGGATTAGATACCCTGGTAGTCCTTGCCGTAAACGATGCATA
7E                 AGCAAGGGGAGCAAACAGGATTAGATACCCTGGTAGTCCTTGCCGTAAACGATGCATA
8E                 AGCAAGGGGAGCAAACAGGATTAGATACCCTGGTAGTCCTTGCCGTAAACGATGCATA
123E               AGCATGGGGAGCAAACAGGATTAGATACCCTGGTAGTCCATGCCGTAAACGATGTATA
28E                AGCATGGGGAGCAAACAGGATTAGATACCCTGGTAGTCCATGCCGTAAACGATGTATA
11E                AGCAAGGGGAGCAAACAGGATTAGATACCCTGGTAGTCCTTGCCGTAAACGATGTATA
120E               AGCAAGGGGAGCAAACAGGATTAGATACCCTGGTAGTCCTTGCCGTAAACGATGTATA
18E                AGCAAGGGGAGCAAACAGGATTAGATACCCTGGTAGTCCTTGCCGTAAACGATGTATA
24E                AGCAAGGGGAGCAAACAGGATTAGATACCCTGGTAGTCCTTGCCGTAAACGATGTATA
39E                AGCAAGGGGAGCAAACAGGATTAGATACCCTGGTAGTCCTTGCCGTAAACGATGTATA
32E                AGCAAGGGGAGCAAACAGGATTAGATACCCTGGTAGTCCTTGCTGTAAACGATGTACA
33E                AGCAAGGGGAGCAAACAGGATTAGATACCCTGGTAGTCCTTGCCGTAAACGATGTATA
21E                AGCAAGGGGAGCAAACAGGATTAGATACCCTGGTAGTCCTTGCCGTAAACGATGTATA
15E                AGCAAGGGGAGCAAACAGGATTAGATACCCTGGTAGTCCTTGCCGTAAACGATGTATA
14E                AGCAAGGGGAGCAAACAGGATTAGATACCCTGGTAGTCCTTGCCGTAAACGATGTATA
142E               AGCAAGGGGAGCAAACAGGATTAGATACCCTGGTAGTCCTTGCCGTAAACGATGTATA
46E                AGCTAGGGGAGCAAACAGGATTAGATACCCTGGTAGTCCTAGCCGTAAACGATGTATA
126E               AGTTAGGGGATCAAACAGGATTAGATACCCTGGTAGTCCTAACCGTAAACGATGTATA
C.pneumoniae_AR39  AGCAAGGGGAGCAAACAGGATTAGATACCCTGGTAGTCCTTGCCGTAAACGATGCATA
                   **   ***** ********** *****************   *  ***** *** * *
```

Figure S3. Alignment of panNPCR 58 nt sequences from blood. Polymorphic sites are red shadowed. "Chlamydia-related bacteria" are grey shadowed

Table S1. Sputum sample data.

Number	Sample	Age	Gender	NPCR 461 bp	mtDNA	NPCR 121 bp	5x2x5 panNPCR
1	DS	27	F	+	+	+	+
2	MŠ	24	F	-	+	-	-
3	BŠ	24	F	-	+	-	-
4	MS	27	M	-	+	-	-
5	PS	60	M	+	+	+	+
6	MR	24	M	+	+	+	+
7	2SP	24	F	-	+	+	+
8	3SP	25	F	-	+	-	-
9	4SP	27	F	-	+	-	-
10	5SP	28	M	+	+	+	nd
11	6SP	55	F	-	+	-	+ [CRB]
12	7SP	58	F	-	+	-	-
13	10SP	27	F	+	+*	+	nd
14	11SP	60	F	-	+	-	-
15	17SP	28	F	-	+	-	-
16	18SP	30	M	+	+	+	nd
17	20SP	26	F	+	+	+	nd
18	21SP	27	F	+	+	+	nd
19	27SP	22	F	-	+	-	+ [CRB]
20	28SP	55	M	-	+	+	nd
21	29SP	35	M	-	+	-	-
22	30SP	53	M	-	+	-	-
23	33SP	23	Ž	+	+	+	nd
24	34SP	27	M	-	+	-	-

25	37SP	49	Ž	-	+	-	-
26	38SP	49	M	-	+	-	-
27	39SP	22	F	-	+	-	-
28	41SP	65	M	-	+	-	-
29	44SP	65	M	-	+	-	-
30	45SP	31	M	+	+	+	nd
31	48SP	53	F	-	+	-	-
32	49SP	72	M	-	+	-	-
33	52SP	29	F	-	+	-	-
34	54SP	22	F	-	+	-	-
35	58SP	26	M	-	+	-	+^{CRB}
36	60SP	22	M	-	+	-	-
37	61SP	26	F	-	+	-	-
38	64SP	23	F	-	+	-	-
39	66SP	20	F	-	+	-	+^{CRB}
40	67SP	20	F	-	+	+	+
41	68SP	26	F	-	+	-	-
42	69SP	40	F	-	+	-	-
43	70SP	31	M	-	+	-	-
44	1002	41	M	-	+	-	-
45	3001	25	M	-	+	-	+
46	3002	25	M	-	+	+	nd
47	3003	25	M	-	+	+	nd
48	3004	24	M	-	+	-	-
49	3005	25	M	-	+	-	-
50	3006	22	M	-	+	-	-
51	3007	28	F	-	+	-	-
52	3008	30	M	-	+	-	-
53	3009	20	M	-	+	-	-
54	3010	22	M	-	+	-	-
55	3011	26	M	-	+	-	-
56	3012	21	M	-	+	-	-
57	3013	28	M	-	+	-	-
58	3014	22	M	+	+	+	nd
59	3015	27	M	-	+	-	-
60	3016	27	M	+	+	+	+
61	3017	30	M	-	+	-	-
62	3018	21	M	-	+	-	-
63	3019	30	M	-	+	-	-
64	3020	30	M	-	+	-	-
65	3021	19	M	-	+	-	+^{CRB}
66	3022	21	M	-	+	+	+
67	3023	24	M	-	+	-	-
68	3024	24	M	-	+	-	-
69	3025	24	M	-	+*	-	-
70	3026	22	M	-	+	-	+^{CRB}
71	3027	21	M	-	+*	-	-
72	3028	26	M	-	+	-	-
73	3029	24	M	-	+	-	-

74	3030	23	F	-	+	-	-
75	3031	26	M	-	+	+	nd
76	3032	21	M	-	+	-	-
77	3033	30	M	-	+	-	+ CRB
78	5002	63	F	-	+	-	-
79	5003	57	F	-	+	-	-
80	5004	51	M	-	+	-	-
81	5005	77	M	-	+	+	+
82	5006	74	F	-	+	-	-
83	5007	66	M	-	+	-	-
84	5008	51	F	-	+	-	-
85	5009	66	F	-	+	+	nd
86	2CHP	24	M	-	+*	-	+
87	3CHP	60	F	-	+	-	-
88	4CHP	41	F	-	+*	-	-
89	5CHP	59	M	-	+*	-	-
90	7CHP	77	M	-	+	+	+
91	9CHP	63	F	-	+	-	-
92	11CHP	29	M	-	+	-	+
93	13CHP	44	F	-	+*	+	nd
94	14CHP	47	M	-	+	+	+
95	15CHP	47	M	-	+*	-	-
96	16CHP	26	F	-	+	+	nd
97	18CHP	28	M	-	+	+	nd
98	19CHP	25	M	-	+	-	+
99	20CHP	53	F	-	+	-	-
100	21CHP	42	F	-	+*	-	+
101	23CHP	27	M	-	+	-	+
102	24CHP	33	F	-	+	-	+
103	27CHP	48	M	-	+	-	-
104	29CHP	44	F	-	+	-	-
105	30CHP	55	F	-	+	-	-
106	31CHP	66	M	-	+	-	+
107	33CHP	69	M	-	+	-	-
108	36CHP	62	F	-	+	-	+
109	37CHP	67	M	-	+*	-	-
110	38CHP	43	M	-	+	-	-
111	39CHP	39	F	-	+	-	-
112	41CHP	61	F	-	+	+	+
113	45CHP	16	F	-	+	+	nd
114	48CHP	37	M	-	+	+	nd
115	49CHP	37	F	-	+	+	+
116	52CHP	30	M	-	+*	-	+ CRB
117	53CHP	55	F	-	+	-	-
118	57CHP	43	F	+	+	+	+ CRB
119	58CHP	67	F	-	+	+	nd
120	60CHP	22	M	-	+*	+	nd
121	62CHP	60	M	-	+	-	-
122	63CHP	27	F	-	+	-	-

123	64CHP	24	F	-	+	-	+CRB
124	71CHP	28	M	-	+	+	nd
125	73CHP	33	M	-	+*	-	+
126	74CHP	56	F	-	+	-	+ CRB
127	76CHP	29	M	-	+	-	-
128	77CHP	33	M	-	+*	-	-
129	78CHP	60	F	-	+	+	nd
130	80CHP	34	M	-	+*	-	-
131	81SP	38	F	-	+	-	+ CRB
132	84CHP	60	F	-	+	+	nd
133	85CHP	61	M	-	+	-	-
134	88CHP	49	M	-	+	-	-
135	91CHP	37	F	-	+	-	-
136	92CHP	43	M	-	+*	-	-
137	93CHP	70	F	-	+	+	nd
138	96CHP	45	F	-	+	-	-
139	97CHP	27	F	-	+*	+	nd
140	98CHP	44	F	-	+*	-	+
141	101CHP	25	F	-	+	-	+
142	102CHP	26	M	-	+*	-	-
143	105CHP	34	F	-	+	-	-
144	107CHP	23	F	-	+*	-	-
145	108CHP	25	F	-	+	-	-
146	109CHP	52	F	-	+	-	+ CRB
147	110CHP	58	M	-	+	-	+
148	111CHP	-	M	-	+	+	+
149	28CHP	32	M	-	-[e]	nd	nd
150	34CHP	33	M	-	+	+[x]	-
151	35CHP	32	F	-	+	+[x]	+[x]
152	70CHP	25	F	-	-[e]	nd	nd
153	75CHP	29	F	-	+	+[x]	+[x]
154	94CHP	45	M	-	+*	+[x]	+[x]
155	95CHP	36	F	-	-[e]	nd	nd
156	104CHP	49	M	-	+	+[x]	+[x]

M male; F; F Female; *1023 bp mtDNA amplicon absent, but 250 bp present; nd not determined; [e] excluded from further analysis due to the DNA degradation. [x] not included due to low PCR yield (DNA was not sequenced or provided unreadable sequence); CRB - DNA sequence from *Chlamydia*-related bacteria".

Table S2. ELISA vs panNPCR in blood

Number	Sample	Gender	Age	IgM	IgG	IgA	mtDNA	5x2x5 panNPCR	Free DNA
1.	1E	M	87	-	+/-10.1	11.8	+	+[C]	+
2.	2E	F	43	-	-	-	+	-	nd
3.	3E	M	56	-	+/-10.1	-	+	-	nd
4.	4E	F	70	-	+/-10.7	-	+	+	nd
5.	5E	F	86	-	-	-	+	-	-
6.	6E	M	53	-	+/-8.6	-	+	+[C]	-

7.	7E	F	35	-	+/-10.9	-	+	+[C]	-
8.	8E	F	43	+13.5	-	-	+	+[C]	-
9.	10E	F	11	-	-	-	+	-	nd
10.	11E	F	28	-	+/-10.6	-	+	+[CRB]	nd
11.	12E	F	52	-	-	-	+	-	nd
12.	13E	M	43	-	+12.7	-	+	-	nd
13.	14E	F	39	+/-11.5	-	-	+	+[CRB]	+
14.	15E	F	29	+12	-	-	+	+[CRB]	nd
15.	16E	M	56	-	+/-8.7	-	+	-	nd
16.	17E	F	54	-	+/-9	-	+	-	nd
17.	18E	M	73	-	+/-9.8	-	+	+[CRB]	nd
18.	19E	F	67	-	+/-11	+/-8.5	+	-	nd
19.	20E	M	62	-	-	-	+	+[C]	nd
20.	21E	F	28	-	-	-	+	+[CRB]	nd
21.	22E	F	73	-	+/-9.9	-	+	-	nd
22.	23E	F	64	-	-	-	+	-	nd
23.	24E	M	64	+15.4	+/-8.9	-	+	+[CRB]	+
24.	25E	F	47	-	-	-	+	-	nd
25.	26E	M	40	-	+/-9.9	-	+	-	nd
26.	27E	F	66	-	-	-	+	-	nd
27.	28E	M	30	-	-	-	+*	+[CRB]	nd
28.	29E	F	65	-	-	-	+*		nd
29.	30E	F	69	-	-	-	+	-	nd
30.	31E	F	60	-	-	+/-11.3	+	-	nd
31.	32E	M	42	+17.5	-	-	+	+[CRB]	+
32.	33E	F	41	+/-8.9	-	-	+	+[CRB]	-
33.	35E	F	81	-	-	+/-11.5	+*	-	nd
34.	36E	F	43	-	+/-9.5	-	+	-	nd
35.	38E	F	44	+/-8.8	-	-	+	+[C]	-
36.	39E	M	46	-	-	-	+	+[CRB]	nd
37.	41E	M	26	-	-	+/-9.8	+	-	nd
38.	42E	F	36	-	-	-	+	+[C]	nd
39.	43E	F	38	-	-	-	+	-	nd
40.	44E	F	45	-	+13.1	-	+*	-	nd
41.	45E	F	41	-	-	-	+	-	nd
42.	46E	M	59	-	+12	-	+*	+[CRB]	nd
43.	47E	M	10	-	-	-	+*	-	nd
44.	48E	F	52	-	-	-	+	+[C]	nd
45.	49E	M	17	-	-	-	+	-	nd
46.	50E	F	35	-	-	-	+	-	nd
47.	51E	F	10	-	-	-	+	-	nd
48.	52E	F	37	-	+/-10.8	-	+	+[C]	nd
49.	53E	F	50	-	+13.2	-	+	-	nd
50.	54E	F	75	-	-	-	+	-	nd
51.	55E	F	90	-	+14.2	+25	+	-	nd
52.	56E	F	42	-	-	-	+	-	nd

53.	57E	F	31	-	+12.2	-	+	-	nd
54.	58E	M	43	-	-	-	+	-	nd
55.	59E	F	5	-	-	-	+	+	nd
56.	60E	F	79	-	+/-11	-	+	-	nd
57.	66E	M	87	-	+12.9	-	+	-	nd
58.	67E	F	67	-	+12.2	-	+*	-	nd
59.	69E	M	35	-	+12.7	-	+*	-	nd
60.	70E	M	64	-	+/-10.7	-	+	-	nd
61.	71E	M	67	-	+14.8	+12.3	+	-	nd
62.	72E	F	66	-	+/-8.7	-	+	+	nd
63.	73E	M	48	-	+13.2	-	+	-	nd
64.	74E	F	66	-	+22.3	+12	+*	-	nd
65.	75E	M	59	-	+23.8	+33.2	+	-	nd
66.	76E	F	37	-	-	-	+	-	nd
67.	77E	F	45	-	-	-	+	-	nd
68.	78E	F	39	-	+12.5	-	+	+	nd
69.	79E	F	31	-	+/-10	+14.3	+	+	nd
70.	84E	F	39	-	+13.4	-	+	+	nd
71.	85E	F	43	-	-	+14.7	+	$+^{CRB}$	nd
72.	86E	M	64	-	+24.8	+12.8	+	-	nd
73.	88E	M	36	-	-	+/-9.4	+	-	nd
74.	89E	F	82	-	-	-	+	-	nd
75.	90E	F	62	-	+/-8.5	+20	+	-	nd
76.	95E	F	17	+/-10.7	-	-	+	-	nd
77.	115E	F	50	+19	+12.5	-	+	-	nd
78.	116E	M	31	+/-10.1	-	-	+	-	nd
79.	120E	F	40	+/-11.5	+14.2	-	+*	$+^{CRB}$	nd
80.	122E	F	73	+/-9	+14.5	-	+	$+^{CRB}$	nd
81.	123E	F	68	+/-10	+/-11.3	-	+	$+^{CRB}$	nd
82.	124E	F	70	-	+20.7	-	+	-	nd
83.	125E	M	46	-	+17.5	-	+	-	nd
84.	126E	M	34	+12.8	+/-10.1	-	+	$+^{CRB}$	nd
85.	127E	M	35	-	+19.9	+/-9.9	+	-	nd
86.	130E	M	72	-	+16.3	-	+	-	nd
87.	131E	F	8	+/-10.1	-	-	+	-	nd
88.	132E	F	39	-	+16.1	-	+	-	nd
89.	137E	F	66	-	+22.7	-	+	-	nd
90.	142E	M	46	+/-10.3	-	-	+	$+^{CRB}$	nd
91.	143E	F	34	-	+15.5	-	+	+	nd
92.	151E	F	26	+/-10.3	-	-	+	-	nd
93.	152E	F	66	+/-10.3	-	-	+	-	nd
94.	153E	M	47	+/-10.3	-	-	+	-	nd

M male; F female; ELISA IgA, IgM, IgG: Cut-off: 10; Grey zone: 8,5-11,5; Negative: <8,5; Positive: >11,5. C - DNA sequence from the genus Chlamydia, CRB - DNA sequence from *Chlamydia*-related bacteria". *1023 bp mtDNA amplicon absent, but 250 bp present; nd - not determined; 4E, 59E, 72E, 78E, 79E, 84E, 143E were not sequenced.
